# Biomechanical Factors in Planning of Periacetabular Osteotomy

**DOI:** 10.3389/fbioe.2013.00020

**Published:** 2013-12-10

**Authors:** Noushin Niknafs, Ryan J. Murphy, Robert S. Armiger, Jyri Lepistö, Mehran Armand

**Affiliations:** ^1^Department of Biomedical Engineering, Johns Hopkins University, Baltimore, MD, USA; ^2^Department of Research and Engineering Development, Johns Hopkins University Applied Physics Laboratory, Laurel, MD, USA; ^3^Department of Mechanical Engineering, Johns Hopkins University, Baltimore, MD, USA; ^4^ORTON Orthopaedic Hospital, Helsinki, Finland

**Keywords:** periacetabular osteotomy, preoperative planning, articular cartilage thickness, cartilage compressibility, biomechanical analysis

## Abstract

**Objective**: This study addresses the effects of cartilage thickness distribution and compressive properties in the context of optimal alignment planning for periacetabular osteotomy (PAO).

**Background**: The Biomechanical Guidance System (BGS) is a computer-assisted surgical suite assisting surgeon’s in determining the most beneficial new alignment of a patient’s acetabulum. The BGS uses biomechanical analysis of the hip to find this optimal alignment. Articular cartilage is an essential component of this analysis and its physical properties can affect contact pressure outcomes.

**Methods**: Patient-specific hip joint models created from CT scans of a cohort of 29 dysplastic subjects were tested with four different cartilage thickness profiles (one uniform and three non-uniform) and two sets of compressive characteristics. For each combination of thickness distribution and compressive properties, the optimal alignment of the acetabulum was found; the resultant geometric and biomechanical characterization of the hip were compared among the optimal alignments.

**Results**: There was an average decrease of 49.2 ± 22.27% in peak contact pressure from the preoperative to the optimal alignment over all patients. We observed an average increase of 19 ± 7.7° in center-edge angle and an average decrease of 19.5 ± 8.4° in acetabular index angle from the preoperative case to the optimized plan. The optimal alignment increased the lateral coverage of the femoral head and decreased the obliqueness of the acetabular roof in all patients. These anatomical observations were independent of the choice for either cartilage thickness profile, or compressive properties.

**Conclusion**: While patient-specific acetabular morphology is essential for surgeons in planning PAO, the predicted optimal alignment of the acetabulum was not significantly sensitive to the choice of cartilage thickness distribution over the acetabulum. However, in all groups the biomechanically predicted optimal alignment resulted in decreased joint contact pressure and improved acetabular coverage.

## 1 Introduction

Numerous outcome studies performed during the last 30 years have shown that performing periacetabular osteotomy (PAO) on young adults with dysplasia is a very effective surgery and prevents or delays osteoarthritis of the hip (Siebenrock et al., 1999; Trumble et al., 1999; Ganz and Leunig, 2007). Typically, surgeons plan the osteotomy on the basis of geometry so that the radiological angles, representing contact surface orientation, reproduce those of normal hips (Wiberg, 1939; Anda et al., 1991; Tallroth and Lepisto, 2006). Several authors (e.g., Hipp et al., 1999; Chao et al., 2000; Mechlenburg et al., 2010; Zhao et al., 2010) have shown that biomechanical planning based on hip geometry corresponds to what surgeons do in practice. However, none of the literature review how the inclusion of biomechanical parameters (especially cartilage thickness) may further affect both planning and the surgical outcome.

The Biomechanical Guidance System (BGS) (Armand et al., 2004, 2005a; Armiger, 2006; Lepistö et al., 2008; Armiger et al., 2009) is a computer-assisted surgical suite for performing PAO. The system combines geometric and biomechanical feedback with intra-operative tracking to guide the surgeon through the PAO procedure. The BGS performs discrete element analysis (DEA) to estimate the contact pressure on a patient-specific model of the joint surface (An et al., 1990). DEA has been shown to approximate the location and magnitude of the peak contact pressure with an accuracy that is not significantly different from that of more computationally expensive finite element methods (Li et al., 1997) in much shorter periods of time, though the distribution is smoother and peak pressures are underestimated.

During contact pressure analysis with DEA, one can consider a variety of biomechanical factors. Choices for modeling the variation of cartilage compressibility determine the DEA type (linear vs. non-linear), and choices for cartilage thickness distribution affect the stiffness matrix computation. Cartilage thickness distribution has been the subject of several studies (Rushfeldta et al., 1981; Hodler et al., 1992; Athanasiou et al., 1994; Nishii et al., 2004, 2005) which have measured thickness maps for acetabular and femoral cartilage over the joint contact surface in normal and dysplastic patients. Nishii et al. (2004) conducted an analysis of magnetic resonance (MR) scans from normal and dysplastic cohorts to define thickness distribution of acetabular cartilage and noted the significant differences between the two cohorts. Several studies have reported on the significance of subject-specific acetabular and femoral geometries (e.g., Anderson et al., 2008, 2010; Lenaerts et al., 2008, 2009; Chegini et al., 2009; Gu et al., 2010), and cartilage thickness and distribution (e.g., Anderson et al., 2008, 2010) to the contact pressures found in the hip joint. Despite the evidence for the significance of subject-specific cartilage thickness variations, these variations have not – to our knowledge – been incorporated into the biomechanical analysis in computerized planning of PAO (Hipp et al., 1999; Armand et al., 2004, 2005b; Armiger et al., 2009).

Several studies (e.g., Abraham et al., 2013) have indicated that loading the hip joint can, in certain circumstances, result in multiple peaks. Based on its formulation, DEA will produce only a single peak in response to a loading profile. However, to our knowledge, no studies have investigated how the single peak produced from DEA impacts biomechanical planning for PAO through correlation with surgical practice.

In this study, we investigate the potential differences between the biomechanically predicted optimal alignment for the acetabular fragment of dysplastic hips under varying cartilage thickness and compressibility models using patient-specific acetabular geometries derived from preoperative CT scans. Specifically: would varying cartilage thickness or the cartilage compressibility model in defining the optimal biomechanical alignment of the hip significantly affect surgeons’ current practice and change the alignment goals of PAO (Hipp et al., 1999; Armand et al., 2004, 2005b; Armiger et al., 2009).

## 2 Materials and Methods

This study was a retrospective evaluation of preoperative CT scans of a cohort of 29 patients (26 female, 3 male) treated with PAO (13 left hip, 16 right hip) under Institutional Review Board approval (JHM IRB1 #05-09-02-01). All patients were diagnosed with developmental dysplasia of the hip and underwent PAO at Orton Hospital in Helsinki, Finland. Nineteen patients were treated between October 1995 and February 1997; the remaining 10 were treated between November 2005 and April 2006. Patients with concurrent pathologies of slipped capital femoral epiphysis and Legg-Calvé-Perthes syndrome were excluded from both PAO and the present study.

**Figure 1:**
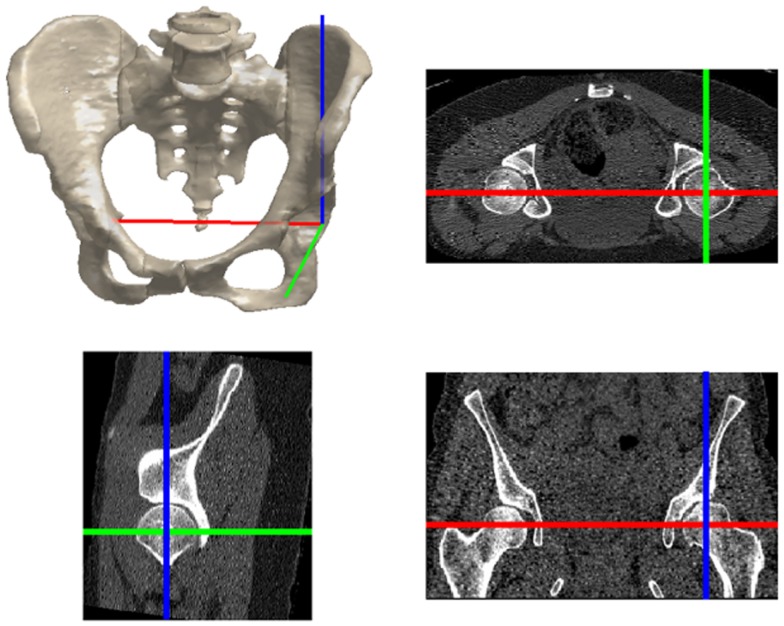
**The hip joint coordinate frame with the X (red), Y (green), and Z (blue) axes pointing from left to right, posterior to anterior, and inferior to superior, respectively**.

Each patient scan was performed in the supine orientation and covered the entire joint region (the acetabulum and proximal femur) in axial slices. Axial slices comprising the scanned volume had variable and sometimes non-uniform spacing; however, the maximum slice spacing was smaller than 1.6 mm in the entire scan volumes and the hip joint was usually scanned at higher resolution compared to the rest of the scan volume. For consistency, the scan volumes were re-sampled at 1 mm spacing and realigned such that the *X* axis contained the centers of the femoral heads (Figure 1).

### 2.1 Joint model creation

**Figure 2:**
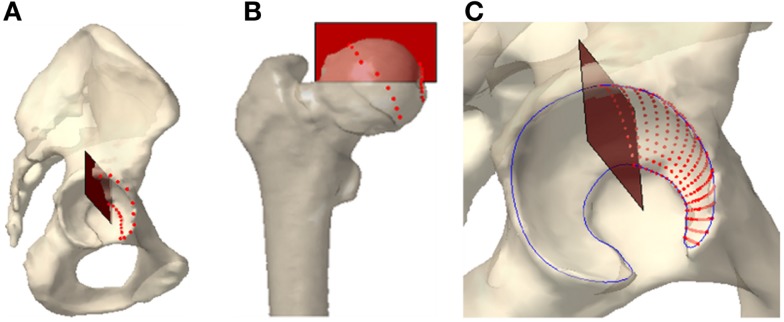
**The Lunate-Trace segmentation technique selects the lateral and medial edges of the contact surface on (A) the acetabulum and (B) the femoral head. (C) Radial and polar interpolation between the edge points yield arc cross sections of the contact surface**.

Since bone morphology has been shown to play a significant role in prediction of cartilage stress (Anderson et al., 2008, 2010; Lenaerts et al., 2008, 2009; Chegini et al., 2009; Gu et al., 2010), we manually extracted subject-specific surface models of the femoral head and acetabulum. These subject-specific, non-spherical surface models were created using the Lunate-Trace algorithm (Armiger et al., 2007), which rotates oblique CT reformats of the hip joint about the medio-lateral axis of the hip (Figure 2). Using this procedure, the acetabular and femoral surfaces were specified as triangular surface meshes. The acetabular surface meshes were composed of a set of 1648 ± 150 triangular elements, each 1.49 mm ± 0.74 mm in area. In previous (unpublished) analysis on the Lunate-Trace algorithm, there was an average area difference of 185.6 mm ± 154.5 mm in the acetabular meshes between two trained users corresponding to an average difference in contact pressures of 0.027 ± 0.24 MPa, indicating minor variability in biomechanical analysis between independently segmented acetabulums. The surface meshes used in the present study were generated by a trained user.

### 2.2 Cartilage representation

We created four distinct cartilage profiles for each contact surface: a uniform thickness profile, population-based normal and dysplastic profiles, and a CT-based profile. All generated acetabular surface models were subject-specific. However, one can assume various models for cartilage thickness distribution over a given acetabular surface. The first profile was a uniformly distributed layer of cartilage, where the biomechanics are independent of the arbitrary thickness value of 2.66 mm (Rushfeldta et al., 1981) used in the simulation.

**Figure 3:**
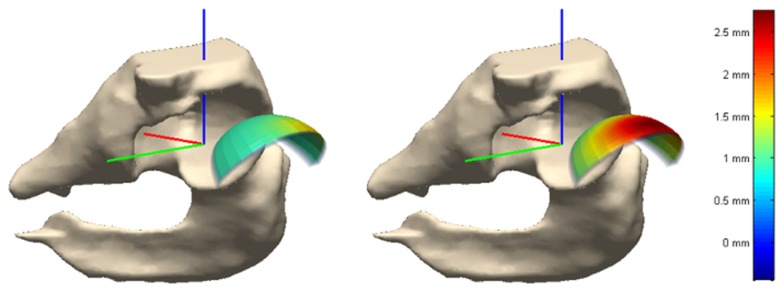
**Cartilage thickness map on weight-bearing area in normal (left) and dysplastic (right) populations**. The weight-bearing area is moved out of the joint for better visualization.

The second and third thickness maps were based on the mean cartilage distribution profile in normal and dysplastic populations (Nishii et al., 2004). The average distribution maps were expressed as the mean cartilage thickness measured over a grid of longitude and latitude across the weight-bearing area (Figure 3). We conducted a two-dimensional Gaussian fit to these data to define models corresponding to the average cartilage thickness maps in dysplastic and normal populations. We aligned the patient-specific acetabular contact surface in a spherical coordinate frame consistent with the Gaussian model. Note that this was not a spherical fit, as the geometries of the dysplastic hips are not spherical. Then, we applied the Gaussian model to the manually segmented contact surface to derive population-based cartilage thickness maps for each patient. Despite the dysplastic nature of the hips in this study, we included both normal and dysplastic population-based cartilage thickness models since recent research has suggested that PAO helps to normalize force distribution (Mechlenburg et al., 2010).

**Figure 4:**
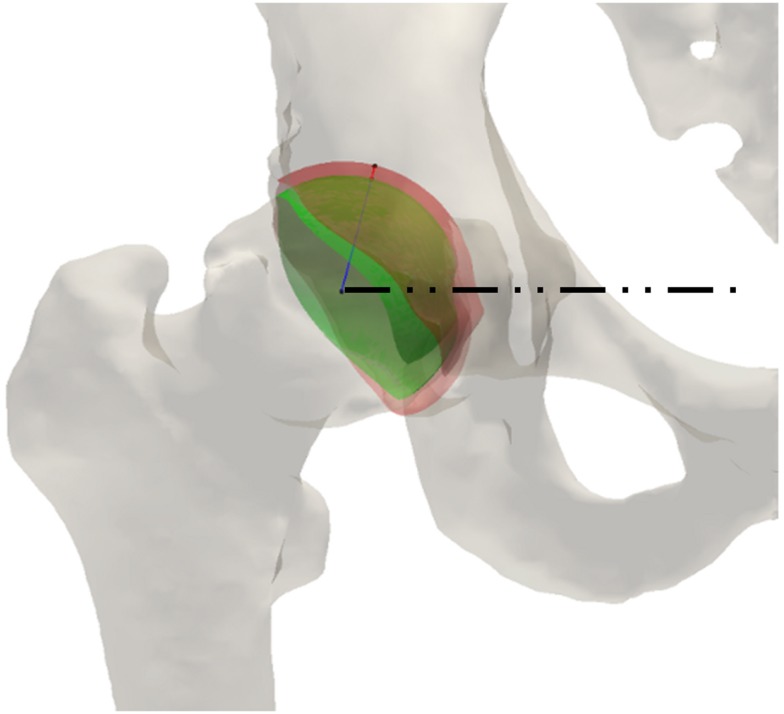
**Ray-firing method for indirect computation of cartilage thickness from CT data using bony surfaces**. The green and red surface models represent the contact surface on femoral and acetabular sides, respectively. The blue line shows the radial direction from the center of the femoral head to an arbitrary point on the femoral head surface. The red line delineates the radial distance between the femoral head and acetabulum contact surfaces. The dashed line is the medio-lateral axis of the hip, extending through the centers of the femoral heads.

The fourth thickness map was an estimate of the subject-specific cartilage thickness. Here, we took the femoral and acetabular contact surfaces to delineate the articular cartilage in the hip joint. The geometry of the hip joint enabled creation of a radial distance map between the femoral and acetabular contact surfaces. We used a ray-firing method from the center of the femoral head to compute the radial distance from element centers of the acetabular surface to the femoral head contact surface (Figure 4). These radial distances comprised the cartilage thickness distribution over the acetabular surface.

### 2.3 Contact pressure computation

Contact pressure distributions over the articular surface were computed by DEA (An et al., 1990; Genda et al., 1995, 2001; Yoshida et al., 2006). The interaction of rigid bony structures through an elastic layer of cartilage was analyzed by modeling the cartilage layer as a set of compression springs over the potential contact area. Each triangular element of the acetabular surface mesh was modeled as a compression spring attached to the center of the corresponding element. Since the elastic modulus of cartilage is much less than that of bone (e.g., Blankevoort et al., 1991), one can assume intra-element deformations are much smaller than inter-element displacements, making DEA an appropriate method for contact pressure calculations (Kawai and Toi, 1981).

The classical formulation of DEA models cartilage as a set of linear compression springs (An et al., 1990). The shear spring constant (*k_s_* = 0.001 N/mm) was much less than the compressive spring constant (*k_d_*) to ensure negligible shear forces over the articular surface (Yoshida et al., 2006). The compressive spring constant *k_d_* was derived on a per-element basis as

(1) $$k_{d}=\frac{E\left(1-\nu \right)A_{i}}{\left(1+\nu \right)\left(1-2\nu \right)h_{i}}$$

assuming Young’s modulus of *E* = 11.58 MPa (Kempson, 1980; Yoshida et al., 2006), Poisson’s ratio of ν = 0.45 (Blankevoort et al., 1991), *A_i_* the area of the specific element, and a *h_i_* the cartilage thickness of a specific element (Athanasiou et al., 1994). Linear DEA is widely used as a first approximation for calculating the contact pressure distribution in cartilage (An et al., 1990; Genda et al., 1995; Schuind et al., 1995; Fregly et al., 2003; Elias et al., 2004; Armiger et al., 2009). Volokh et al. (2007) previously investigated the limits of applicability of such a linear spring model and compared the non-linear axial stress-stretch law to experimental and analytical tests on one-dimensional confined compression of articular cartilage as reported by Ateshian et al. (1997) and Huang et al. (2005). Non-linear DEA inherently models the behavior of cartilage with greater detail and at a higher level of accuracy than classical, linear DEA (Volokh et al., 2007). Non-linear DEA models cartilage compression with non-linear springs to better match the stress-strain curve. Computationally, non-linear DEA employs a Taylor-series expansion of the Cauchy stress:

(2) $$\sigma \left(\varepsilon \right)=\frac{H_{A0}\left({\left(1+\varepsilon \right)}^{2}-1\right)}{2{\left(1+\varepsilon \right)}^{2\beta +1}}\exp\beta \left({\left(1+\varepsilon \right)}^{2}-1\right)$$

about zero strain, ε = 0, where *H_A_*_0_ and β are material properties. In the present work, we used both linear and non-linear DEA to represent variations of cartilage compressibility.

### 2.4 Joint loading conditions

The contact pressure profile over the articular surface was found while applying forces corresponding to daily activities of walking, sitting, and standing. The force pertaining to walking represents the peak of the reported forces over a gait cycle, while the sitting and standing forces denote steady state values. The activity forces – reported as percentage of body weight – were borrowed from previously published work on joint forces of patients with endoprostheses (Bergmann et al., 2001). A constant body weight of 74 kg (163 lbs) was assumed for all patients to remove any scaling effect of body weight on the absolute value of the contact pressure.

### 2.5 Optimization of acetabular surface orientation

The BGS computes the biomechanically optimal alignment of the acetabulum by searching the space of geometrically reasonable orientations of the contact surface (Armiger, 2006; Armiger et al., 2009; Murphy, 2010). The search space includes realignment rotations of ±45° in the sagittal plane, ±60° in the frontal plane, and ±45° in the axial plane. The optimal realignment transformation minimizes the sum of squared peak contact pressures computed for mechanical forces pertaining to daily activities of walking, sitting, and standing. An optimization routine based on a variant of the Levenberg-Marquardt algorithm for non-linear systems in the Matlab^®^ Optimization Toolbox (The MathWorks, Inc. Natick, MA, USA) finds this optimal alignment. As this routine uses only peak contact pressures, the known underestimation and smooth pressure distribution obtained through DEA has no effect. We found the biomechanically optimal alignment of the acetabulum under combinations of different cartilage thickness profiles and models for variation of cartilage compressibility (modeled as a linear spring, linear DEA, or a non-linear spring, non-linear DEA).

### 2.6 Measures of variation

We found the biomechanically optimal alignment of the acetabulum for each patient using all possible combinations of the DEA technique (linear or non-linear) and cartilage thickness map (uniform, based on the normal or dysplastic population, and derived from CT). For each patient, we computed the peak contact pressure in the optimal alignments for the three daily activities (walking, sitting, and standing) and compared those with the preoperative peak contact pressure.

**Figure 5:**
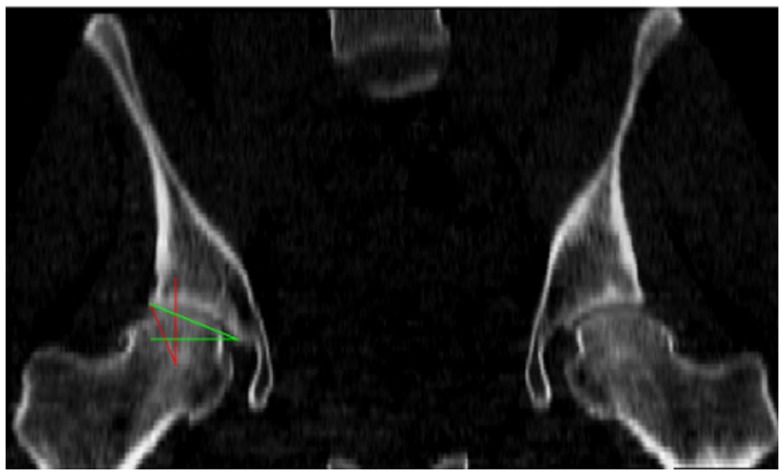
**The radiological angles of the hip include the center-edge (CE) angle, in red, which evaluates the lateral coverage of the femoral head by the acetabulum, and the acetabular index (AC) angle, in green, which represents the obliqueness of the acetabular roof**.

To characterize anatomical variations of the optimal alignments, we used the center-edge (CE) (Wiberg, 1939) and acetabular index (AC) (Tönnis, 1987) angles, which are commonly used radiological metrics for evaluation and surgical treatment of acetabular dysplasia (Figure 5). We evaluated the original and optimized alignments of the acetabulum in terms of satisfying the well-established acceptable ranges for these angles (Tallroth and Lepisto, 2006). We used the method of Armiger et al. (2007) to automatically determine these radiological angles for the original and optimal alignments of the acetabulum. We also compared the realignment rotations based on their rotation components in the different anatomical planes.

### 2.7 Statistical analysis

Analysis of variance (ANOVA) was used to compare the relative decrease of peak pressure – from preoperative to optimal – in combinations with different cartilage thickness and compressive properties. In the event ANOVA testing exhibited significant differences among groups, we used Tukey’s HSD (Honestly Significant Difference) test to determine which group(s) were significantly different. Two series of analyses were carried out to test for significant effects from the two factors: cartilage thickness map and compressive properties.

To study the effect of different cartilage thickness models, results with the same cartilage map but different DEA techniques were combined and the four groups were statistically compared. The influence of cartilage compressive properties was evaluated by combining all results from each DEA technique and comparing the two groups (linear or non-linear DEA). We chose *p*-values smaller than 0.05 to indicate statistical significance.

## 3 Results

**Figure 6:**
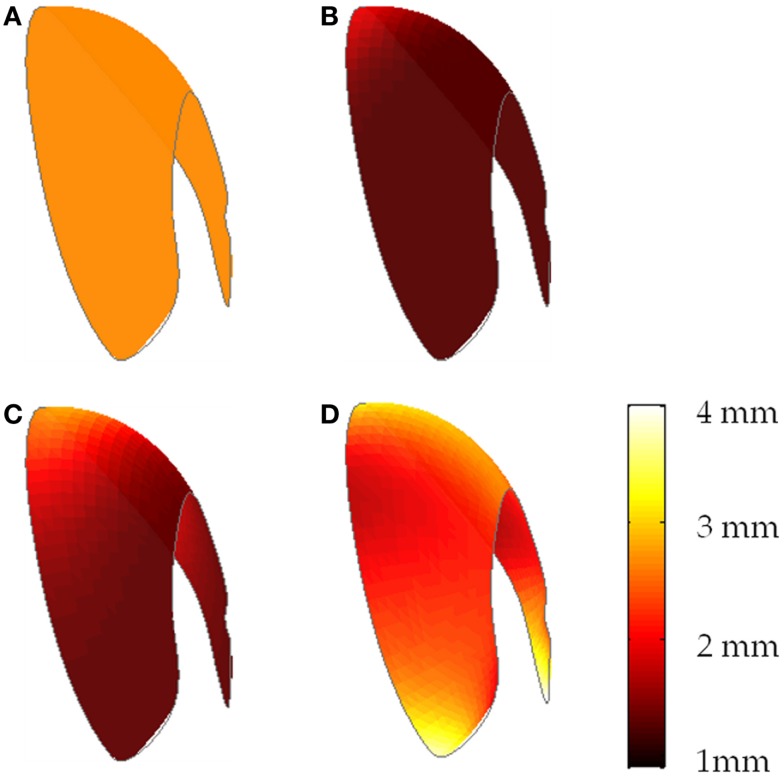
**Cartilage thickness distributions over a sample acetabular surface (frontal view) for (A) uniform thickness of 2.66 mm, (B) population-based normal map, (C) population-based dysplastic map, (D) CT-based map**.

The generated acetabular contact surfaces for each of the four algorithms were visually distinct (Figure 6). In the normal population-based model, cartilage thickness values varied between 1.24 and 1.95 mm; however, in the dysplastic population-based model, thickness values were increased and varied between 1.29 and 2.87 mm. The increased cartilage thickness in the inferior portion of the acetabulum in the CT-based profile was likely an artifact of the ray-firing method. Since the inferior part of the acetabulum did not contribute to weight-bearing, these artifacts did not affect the contact pressure analysis.

In all cases, there was a relative decrease in peak contact pressure for all combinations of cartilage thickness distribution and compressive properties (Tables 1 and 2). On average, the optimal alignment decreased the peak contact pressure by 49.2 ± 22.3%. We found cartilage models to be significantly different from each other when comparing the relative decrease in peak pressure (*p* < 0.001). Specifically, the population-based dysplastic varied from the uniform and the CT-based, and the population-based normal varied from the CT-based. However, there was no significant difference between the linear and non-linear DEA techniques (*p* = 0.66).

**Table 1:** **Relative decrease in peak contact pressure (%) between preoperative and optimized alignments (“p-b” denotes a population-based model)**.

Calculation method	Cartilage thickness model				Mean
	Uniform	p-b Dysplastic	p-b Normal	CT- based	
Linear DEA	52 ± 19	42 ± 22	48 ± 21	55 ± 16	50 ± 20
Non-linear DEA	51 ± 23	35 ± 24	45 ± 23	62 ± 16	48 ± 24
Mean	52 ± 21	39 ± 24	47 ± 22	59 ± 16	49 ± 22

**Table 2:** **Relative decrease in peak contact pressure data (“p-b” denotes a population-based model)**.

Patient	Linear springs				Non-linear springs			
	Uniform	p-b Dysplastic	p-b Normal	CT-based	Uniform	p-b Dysplastic	p-b Normal	CT-based
1	0.56	0.48	0.54	0.57	0.57	0.31	0.50	0.62
2	0.86	0.84	0.86	0.85	0.87	0.85	0.87	0.89
3	0.60	0.45	0.55	0.63	0.61	0.42	0.52	0.65
4	0.65	0.54	0.60	0.67	0.67	0.46	0.56	0.72
5	0.65	0.52	0.59	0.57	0.66	0.50	0.56	0.54
6	0.91	0.89	0.90	0.91	0.92	0.89	0.91	0.92
7	0.58	0.46	0.55	0.64	0.58	0.35	0.54	0.72
8	0.38	0.17	0.29	0.34	0.35	0.07	0.22	0.32
9	0.56	0.42	0.53	0.56	0.59	0.29	0.51	0.56
10	0.59	0.45	0.51	0.58	0.61	0.34	0.49	0.57
11	0.59	0.44	0.54	0.55	0.58	0.39	0.49	0.64
12	0.71	0.65	0.68	0.69	0.74	0.60	0.67	0.73
13	0.65	0.59	0.63	0.64	0.67	0.46	0.62	0.69
14	0.56	0.40	0.52	0.64	0.59	0.29	0.50	0.78
15	0.61	0.55	0.59	0.68	0.63	0.48	0.60	0.90
16	0.63	0.53	0.61	0.64	0.65	0.47	0.59	0.70
17	0.75	0.72	0.73	0.73	0.76	0.68	0.74	0.74
18	0.26	0.06	0.20	0.28	0.19	0.01	0.10	0.50
19	0.38	0.21	0.30	0.30	0.34	0.13	0.24	0.28
20	0.39	0.28	0.34	0.53	0.32	0.30	0.25	0.63
21	0.19	0.04	0.15	0.27	0.11	−0.03	0.07	0.23
22	0.54	0.39	0.52	0.60	0.53	0.37	0.47	0.66
23	0.14	0.03	0.12	0.34	0.07	0.05	0.06	0.63
24	0.57	0.46	0.51	0.57	0.58	0.45	0.47	0.60
25	0.61	0.56	0.61	0.59	0.59	0.52	0.59	0.62
26	0.22	0.07	0.16	0.33	0.14	0.01	0.08	0.63
27	0.42	0.31	0.35	0.45	0.40	0.08	0.26	0.50
28	0.12	0.13	0.07	0.39	0.05	−0.01	0.05	0.50
29	0.62	0.55	0.61	0.61	0.61	0.45	0.57	0.63

**Figure 7:**
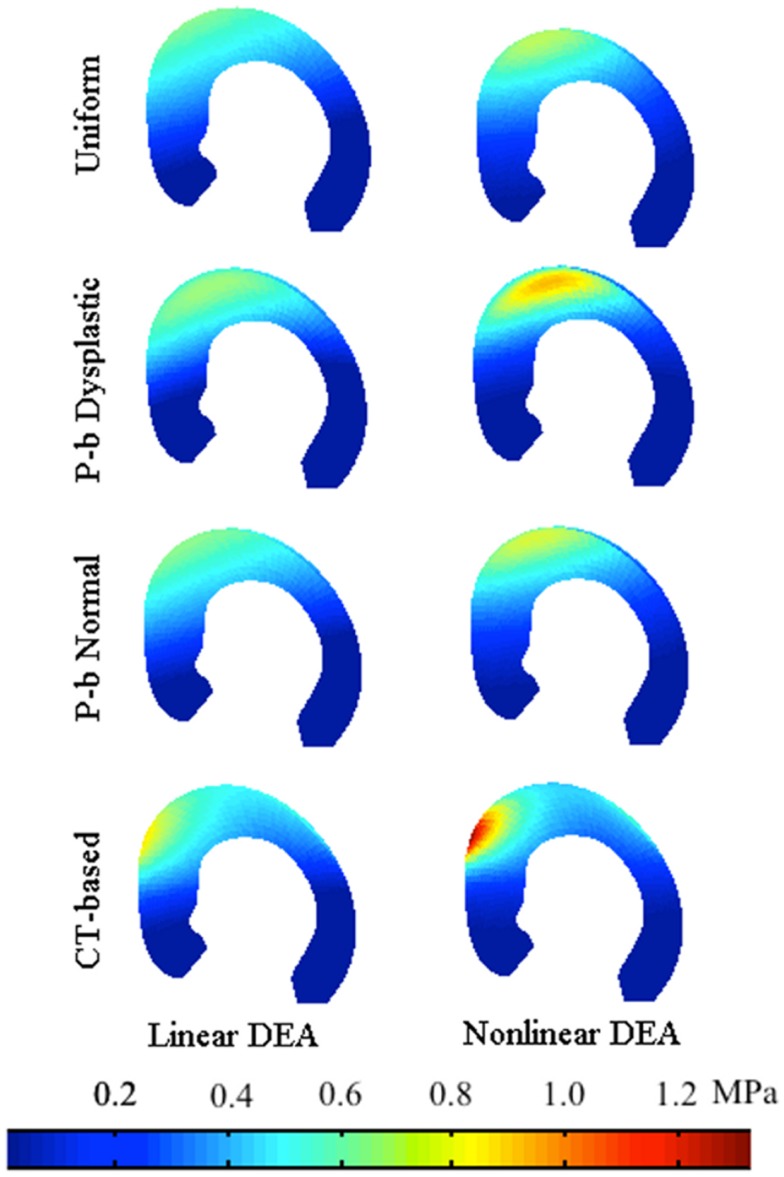
**Pressure profile of a sample acetabulum loaded by the standing force (medial view)**. In this subject, the non-linear DEA-predicted higher stress levels over the acetabulum.

**Figure 8:**
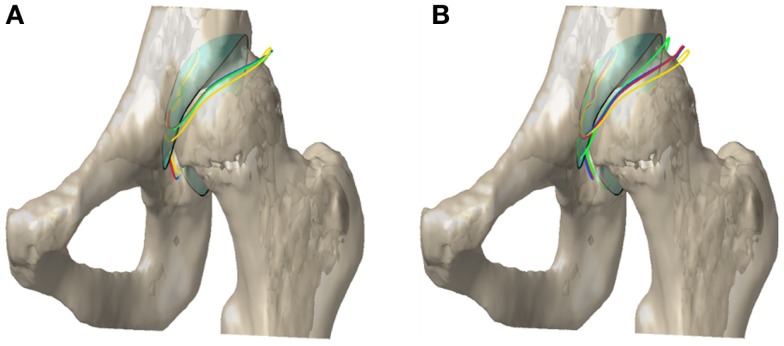
**Acetabular contact surface outline in the original and optimal alignment for (A) linear and (B) non-linear DEA**. The colors red, blue, green, and yellow correspond to uniform, population-based (p-b) normal, p-b dysplastic, and CT-based cartilage thickness models, respectively. The cyan surface with the black outline represents the original alignment of the contact surface.

In almost all patients, the value of maximum contact pressure exhibited a decrease from the original to the optimal alignment (Table 2); however, two patients exhibited a slight increase in the value of maximum pressure (less than 3%) when optimal alignment was found using non-linear DEA with the population-based dysplastic cartilage distribution. On average, the CT-based cartilage thickness profile resulted in the largest decrease in peak contact pressures (59 ± 16%), followed by uniform (52 ± 21%) and population-based cartilage models (47 ± 22% for the normal population and 39 ± 24% for the dysplastic population); i.e., CT-based cartilage models predicted alignments corresponding to the largest predicted benefit in alleviation of contact pressures. A sample pressure profile is illustrated in Figure 7 and a sample realignment is presented in Figure 8.

**Figure 9:**
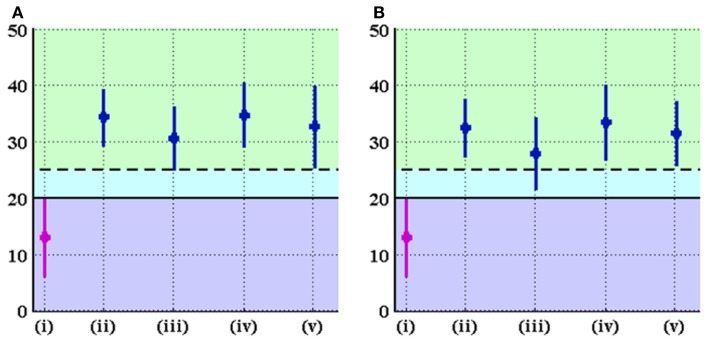
**CE angle (in degrees) in the original and optimal orientations of the acetabulum for (A) linear and (B) non-linear DEA**. The graph displays mean ± standard deviation of measurements for each group. The groups denoted by (i), (ii), (iii), (iv), and (v) represent the original alignment and the optimal alignments found using uniform, p-b dysplastic, p-b normal, and CT-based cartilage thickness models, respectively. The solid line marks the border between the dysplastic and borderline values of the CE angle, while the dashed line represents the border between borderline and normal values of the CE angle. Optimal orientation of the acetabulum results in improvement (increase) of CE angle.

**Figure 10:**
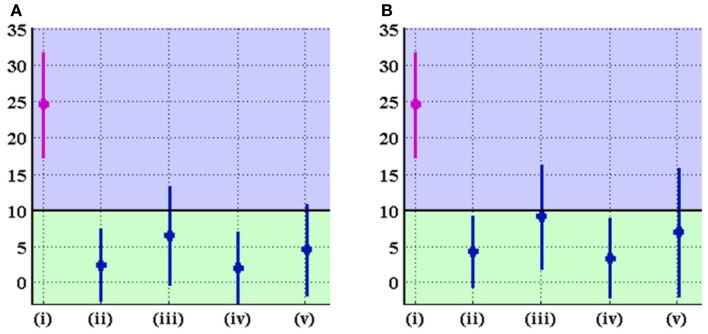
**AC angle (in degrees) in the original and optimal orientations of the acetabulum for (A) linear and (B) non-linear DEA**. The graph displays mean ± standard deviation of measurements for each group. The groups denoted by (i), (ii), (iii), (iv), and (v) represent the original alignment and the optimal alignments found using uniform, p-b dysplastic, p-b normal, and CT-based cartilage thickness models, respectively. The black line marks the border between normal and dysplastic values for the AC angle. Optimal orientation of the acetabulum results in improvement (decrease) of AC angle.

Different cartilage thickness models and DEA techniques resulted in similar improvements in radiological angles, all of which saw increased lateral coverage of the femoral head in the optimized location. Across the eight combinations of cartilage model and DEA technique, the average increase in CE angle from preoperative to optimal was 19.0 ± 2.2°, ranging from 14.7° to 21.5° (Figure 9). Similarly, the average decrease in AC angle was 19.5 ± 2.4°, ranging from 15.3° to 22.4° (Figure 10). Of the 22 cases with CE angle in the dysplastic range, 91.5% moved to normal range (CE >25°) and 8.5% moved to borderline range (20° < CE < 25°) after realignment.

## 4 Discussion

Clinically, PAO is expected to alleviate pain associated with acetabular dysplasia by reducing joint contact pressure (Hipp et al., 1999; Leunig et al., 2001). Here, the relative decrease in maximum contact pressure from preoperative to optimal alignment was chosen as a metric describing how well a combination explains the effectiveness of acetabular realignment. In addition to the relative decrease in contact pressure, we studied the rotations which put the acetabular contact surface in the optimal alignment. We used these measures of variation to evaluate the role of cartilage thickness profile and DEA technique in the optimal alignment planning task in PAO.

As expected, all cartilage thickness distributions decreased the peak contact pressure from original to optimal alignment (Table 1). The CT-based cartilage distribution – which captures patient-specific cartilage geometry – predicted alignments resulting in the largest relative decrease in peak contact pressure. We speculate this may be due to the typical thickening of the cartilage seen at the lateral edge of the dysplastic acetabulum, which is no longer in the direct path of the load after realignment.

The optimal alignments from each thickness model showed, as expected, increased lateral coverage as defined by an increase in the CE angle and a decrease in the AC angle. However, the realignment rotations pertaining to the population-based dysplastic model increased the lateral coverage of the femoral head to a lesser degree compared to others. Of all 58 optimal alignments based on this thickness profile (with linear and non-linear DEA), 15 cases had CE angles below the normal range (CE <25°). We speculate this may be due to the different thickness distribution in the dysplastic population; i.e., the abnormal acetabular configuration in dysplasia exposes the joint surfaces to elevated levels of contact pressure. This may elicit the body to provide more cartilage cushioning (i.e., greater cartilage thickness) over the weight-bearing area as a compensatory mechanism, which is consistent with the observations of Nishii et al. (2004). The thicker layer of articular cartilage will lower the contact pressure in dysplastic patients.

Our study has several limitations, including the technique to measure cartilage thickness on CT scans. CT arthrograms or MRI can be utilized to enhance contrast of the cartilaginous tissue (Chegini et al., 2009) and potentially increase the accuracy of the predicted optimal alignment of the acetabulum. However, the use of these modalities was not part of the existing clinical protocol for performing PAO at Orton Hospital. Moreover, the purpose of the study was to investigate if cartilage thickness would have a clinical impact on the biomechanically optimal alignment of the acetabulum. The results suggest that the optimal alignment was not significantly sensitive to the cartilage thickness profile.

Application of activity forces available from the literature (Bergmann et al., 2001) to the present joint models would yield more accurate results if the models were transformed to a consistent coordinate frame with the forces. Such transformations could not be defined for 19 subjects due to partial coverage of pelvis in their scan volumes. Therefore, forces were applied an intermediate coordinate frame. For 10 subjects where the entire pelvis scan was available, we compared two coordinate frames defined with or without the L5-S1 landmark. Among the 10 subjects, the adjusted supine frame differed from the accurate (Bergmann) coordinate frame by 8.0 ± 4.6°. In the hips where we could define the Bergmann frame, there was a 0.5 ± 0.35 MPa difference in contact pressure between the supine and Bergmann frame. Moreover, a paired *t*-test between contact pressure in the Bergmann frame and those in the adjusted supine frame (that used in this study) showed no significant difference between the data (*p* = 0.464). In addition, it is important to note that using the supine frame as an estimate of the standing frame is not an infrequent clinical practice and has also been used in radiological analysis of the hip (e.g., Genda et al., 2001; Armand et al., 2005b). We, therefore, believe our study makes good use of valuable, available data.

Studies as early as the one by Greenwald and Haynes (1972) have shown that, under normal stance loads, generally the entire articular surface of the acetabulum gets involved in bearing the load. Therefore, pressure is sometimes distributed over a relatively large area with more than one peak point similar to the results of Widmer et al. (2002). More detailed experiments and analyses show that, among the load-bearing areas, anterior and posterior aspects of the acetabulum see the peak pressure (Day et al., 1975; Sparks et al., 2005). Pressure patterns can sometimes resemble a band or a “ridge” rather than a simple peak, and in less frequent occasions can include double peaks (Brown and Shaw, 1983; Bay et al., 1997; von Eisenhart-Rothe et al., 1997; Brand et al., 2001). The observed pressure patterns are dependent on several factors including the variations in the cartilage thickness, surface incongruity of the two contacting bones, and the amount and direction of loading (Bay et al., 1997; Sparks et al., 2005). The nature of our DEA analysis, similar to the model proposed by Yoshida et al. (2006), does not produce multiple peak pressure profiles. However, DEA achieves a first-order estimate of the pressure profile, which does include the critical superolateral pressure (near the edge of the weight-bearing zone) of interest when correcting dysplastic hip joints. Optimizing the orientation of the acetabular surface based on DEA-predicted pressures with single peaks (typically in the superolateral region) resulted in improvements in the radiological angles and the femoral head coverage. These improvements are compatible with the expected outcome of the surgery, encouraging the use of our DEA method for PAO optimization.

In conclusion, while patient-specific acetabular morphology is essential in planning PAO, the predicted optimal alignment of the acetabulum was not significantly sensitive to the choice of cartilage thickness distribution over the acetabulum. Moreover, each group showed clinical improvement in terms of decreasing contact pressures, increasing the lateral coverage, and decreasing the acetabular roof obliqueness. Therefore, through analysis of our patient-specific CT-based cartilage thickness profile, it appears that the additional cost and effort associated with the use of complex and non-uniform cartilage thickness profiles does not provide a great benefit for planning PAO. Moreover, the first-order estimation of the pressure profile using DEA does not appear to interfere with the clinical improvement seen when planning on peak pressure alone. However, future studies including more accurate MRI-based, patient-specific cartilage profiles can potentially present a more thorough argument. Furthermore, we investigated the effect of the constitutive model for cartilage compressibility. We found no significant difference between linear and non-linear DEA techniques by looking at the alleviation of contact pressures. However, similar to our observations regarding the cartilage thickness profiles, the rotational differences between linear and non-linear DEA techniques were less than seven degrees, minimizing their significance in clinical implications (i.e., surgical limitations in realization of the preoperative plan are likely to be a restricting factor on the precision).

## Conflict of Interest Statement

The authors declare that the research was conducted in the absence of any commercial or financial relationships that could be construed as a potential conflict of interest.
